# Validation of the French version of COHIP-SF-19 among 12-years children in New Caledonia

**DOI:** 10.1186/s12903-022-02370-4

**Published:** 2022-08-18

**Authors:** Amal Skandrani, Nada El Osta, Hélène Pichot, Caroline Eschevins, Bruno Pereira, Stéphanie Tubert-Jeannin

**Affiliations:** 1 Centre de Recherche en Odontologie Clinique (CROC), Faculté de chirurgie dentaire, Université Clermont Auvergne, 2 rue de Braga, 63000 Clermont-Ferrand, France; 2 Health and Social Agency of New Caledonia (ASS-NC), Nouméa, New Caledonia France; 3 CHU of Clermont-Ferrand, Clinical Research and Innovation Direction (DRCI), 63003 Clermont-Ferrand, France

**Keywords:** Oral health, Quality of life, Child, Validation study, Dental caries, Socio-economic factors, New Caledonia

## Abstract

**Background:**

Assessment of oral health-related quality of life is now associated to clinical indicators in epidemiological studies. This study aimed at validating the French Short Form of the Child Oral Health Impact Profile (COHIP-SF-19) and assessing the impacts of oral diseases among schoolchildren in New Caledonia (NC).

**Methods:**

A sample of 12-years-old children (n = 971) was selected in 2019 in NC using a random, stratified, and clustered sampling technique. Children filled the French COHIP-SF-19 questionnaire. Information on sociodemographic characteristics, oral hygiene habits, perception of oral health problems were also collected through self-administered questionnaires or from the schools’ database. Dental status (dental caries, gingival status, and dental functional units) was clinically recorded at school by four calibrated examiners. Cronbach’s alpha and intraclass correlation coefficients (ICC) were calculated. Kruskal–Wallis tests and spearman correlations were used along with multilevel mixed models taking into account the cluster and examiner effects. A confirmatory factor analysis was conducted and sensitivity analyses were performed.

**Results:**

Among the 693 children examined, 557 children were included. Oral diseases were frequent in the study population 40% had dental caries and 55% presented gingivitis. The COHIP scores ranged from 7 to 76 (57.9 ± 9.96) with 96.4% of the children having experienced oral health problems, 81.7% reporting functional impacts and 90.5% socio-emotional impacts. Overall, the French COHIP-SF-19 showed satisfactory psychometric characteristics. Internal consistency was high (Cronbach’s alpha = 0.80) and reproducibility excellent (ICC = 0.9). Discriminant and concurrent validity were adequate. Indeed, children with less optimal social situation, impaired dental status, declaring severe dental problems or difficulties in accessing oral health care showed lower COHIP-SF-19 scores. Factor analyses suggested a four-component structure with identification of a new domain (self -image) and changes in the repartition of the items within the original domains. Sensitivity analyses showed similar results for children with partial or complete answers in the COHIP questionnaire.

**Conclusions:**

The French COHIP-SF-19 showed satisfactory psychometric characteristics and allowed to identify the high impacts of oral diseases in New Caledonian children, namely for socially deprived children.

**Supplementary Information:**

The online version contains supplementary material available at 10.1186/s12903-022-02370-4.

## Background

Oral diseases such as untreated dental caries affects 2.4 billion people worldwide, starting early in childhood and disproportionately affecting socially disadvantaged populations. These inequalities persist into adolescence and adulthood, with a cumulative process that, in the absence of prevention or treatment, accentuates the social gradient over time [1–3]. Oral diseases have a significant impact on the physical, social and emotional well-being [4, 5]. The assessment of oral health-related quality of life (OHRQoL) has become increasingly popular for evaluating and consequently planning oral health promotion interventions. As children and adolescents have specific quality of life issues, various instruments have been developed to measure OHRQoL in paediatric populations over the last decades [6–9]. Some of them, such as the Child-OIDP and the COHIP-34 have already been translated and validated in France and namely in New Caledonia [10, 11].

New Caledonia (NC) is a French, south pacific territory which population is highly impacted by “Non Communicable diseases” (NCDs) such as dental caries. NC is characterised by large health inequalities related to social determinants, the province of living (North, South, Loyalty Islands) or to ethnicity. Indeed, the population of NC is multi-ethnic with 41% of the population belonging to the indigenous Kanak community [12, 13].

In 2011–2012, a study assessed the dental status of 6-, 9- and 12-years-old children in NC and showed high levels of caries prevalence while confirming geographical, social and ethnic health disparities [14]. Following this study, an Oral Health Promotion program (OHP) was developed in connection with the prevention of other chronic diseases such as obesity [15]. A second epidemiological survey was conducted in 2019/2020 to appreciate the evolution of the children’s dental status and evaluate the impacts of the OHP program.

The French COHIP-34 scale was chosen to assess OHrQOL in NC as it includes questions that explore the positive aspects of OHRQoL such as confidence or attractiveness. The COHIP-SF-19 is a shortened version of the COHIP scale that is less time-consuming and thus facilitates data collection in large-scale surveys [16]. The COHIP-SF-19 has already been translated into Arabic, Dutch, Japanese and Chinese (Mandarin), which makes it a good research tool for international comparisons. Hence, validating the French version of this short scale is of interest, namely in NC where OHrQOL indicators are needed for monitoring the impacts of the OHP program.

The main objective of this study was to validate the French COHIP-SF-19. Secondly, through the validity analysis, this study also assessed the impacts of oral diseases in New Caledonian schoolchildren.

## Methods

### COHIP-SF-19 questionnaire

The French COHIP-SF-19 questionnaire is derived from the validated French version of the COHIP-SF-34 questionnaire, by selecting 19 items of the English COHIP-SF-19 questionnaire [9, 10, 16].

The French COHIP-SF-19 questionnaire includes 19 questions (items) forming three conceptual subscales: oral health (5 items), functional well-being (4 items), and socio-emotional well-being (10 items). Children are also asked about the frequency with which they have experienced impacts of oral diseases since school year started. Each question can be answered with a five-point Likert scale (4 to 0) ranging from “never", "almost never", "sometimes", "quite often", and "almost always". Two of the questions are positively worded questions, with a reversed scale (0 to 4) where a higher frequency indicates an improved oral health. The COHIP-SF-19 scale (and related sub-scores) is an additive score, varying from 0 to 76, with a low score reflecting an impacted oral health quality of life [16].

### Study population

This study is part of a national epidemiological survey conducted in NC in 2019/2020 among 6, 9 and 12 years old children. The required sample size (N) was calculated to ensure the precision of caries prevalence estimate. Calculations integrated the cluster effect and an estimated participation rate of 85%: At least 970 12-year-old children needed to be selected [14]. This number was adequate for evaluating psychometric properties according to the COSMIN recommendations [17]. The sampling method was similar to the one used for a previous study conducted in 2012 [14]. A random, stratified, and clustered sampling technique was used. The study population was stratified according to the region, the area and school type to ensure representativeness in terms of cultural, ethnic, geographical and social diversity. Clusters were made up of secondary schools and were randomly selected with proportional probability to the size of the cluster. A sample of 971 children was selected from the 3894 12-year-olds recorded by NC educational services.

### Data collection

Ethical approval was obtained from the NC Ethics Committee (Notice 2019-06 002 of June 24th 2019), information letters and consent forms were sent to parents. Only children attending schools that agreed to participate in the study, returned a signed parental consent form and gave verbal agreement were included in the study. The questionnaire was self-administered. Children who responded to less than 75% of the questions per domain were excluded from the analysis. When less than 25% of the responses per domain were missing, missing values were replaced with the mean score of available items.

Children also answered a self-administered questionnaire with socio-demographic variables (gender, ethnicity, place of living…) and questions relating to oral health behaviours (tooth-brushing frequency), perception of oral health problems as well as difficulty for accessing oral health care. Some information were directly retrieved from the school administrative databases (region, health insurance, type of school…).

Dental status was recorded by four calibrated dentists during a clinical oral examination. Caries experience was assessed using ICDAS criteria [18]. Caries prevalence was appreciated with the percentage of children with at least one untreated, filled or missing permanent tooth due to decay (D_3_MFT > 0). The threshold for caries detection was the presence of dentinal lesions (ICDAS 4-6). The Gingival index of Löe and Silness was used to record the presence gingival inflammation and the scores were dichotomized (score 0 for all sextants vs score > 0 for at least one sextant) [19]. In addition, the presence of an infectious process (abscess, tooth with pulpal exposure, fistula), the number of posterior functional units (number of mandibular premolars and molars in occlusion) were recorded [6, 18]. The study variables are presented in Additional file 1: Table S1.

### Data analysis: psychometric testing of the scale

The psychometric properties of COHIP scale were evaluated according to the COSMIN guidelines to meet the main objective of the study [17]. The COSMIN checklist is available in Additional file 5.

Acceptability was evaluated with descriptive statistics of the distribution of COHIP scores and for each item. In addition, floor and ceiling effects (> 15% of the respondents with the lowest or highest score) were identified [20].

Internal consistency was assessed by calculating Cronbach’s alpha coefficient and Cronbach’s alpha if an item was deleted for the overall score and for each of the three sub-scores; a coefficient ≥ 0.7 was considered to indicate satisfactory internal consistency [21]. The item-rest correlation test was performed to check the homogeneity of the scale. Values below 0.2 indicated that the corresponding item did not correlate well with the scale [22].

A confirmatory factor analysis (CFA) with the maximum-likelihood estimation was conducted to confirm factor loading of the COHIP subscales [23]. CFA was undertaken to assess two hypothesized measurement models based on the original COHIP-SF-19 (a three-factor model) and findings of previous exploratory factor analyses (a four-factor model) [16, 24, 25]. In order to obtain the model that best fits the theoretical and quality criteria, a third model resulting from the modification of the first two was also evaluated. A good model fit was evaluated using several indices including the ratio of χ2 to degrees of freedom (χ2/df) with a recommended range of 1.0 to 3.0. A root mean square error of approximation (RMSEA) values ≤ 0.05 indicate good fit. Values between 0.05 and 0.08 indicate reasonable fit. For the incremental fit statistics (The Goodness of Fit Index: GFI; Adjusted Goodness of Fit Index: AGFI; and the comparative fit index: CFI) values above 0.90 and 0.95 indicate reasonable to good fit. The model with the minimum Akaike Information Criterion (AIC) value is regarded as the best fitting model [26].

Reproducibility was checked by calculating the intraclass correlation coefficients (ICC) for the global scores and sub scores. Due to COVID pandemic, it was not possible to re-examine the children. Thus, data from the 2012 test–retest evaluation of the French COHIP-34 were used. An ICC value of at least 0.7 is recommended as a minimum reproducibility standard [20].

Concurrent validity was assessed by examining the relationship between COHIP-SF-19 scores and the rating of self-perceived dental problems, and difficulty for accessing dental care [20]. As the scores were not normally distributed, Kruskal–Wallis tests were used. Effect size was also calculated to highlight the magnitude of the gap between variable categories following Cohen’s recommendations [27]. Then, coefficients of Spearman correlations between those variables and COHIP scores were calculated [20, 28].

Discriminant validity was tested by comparing the mean COHIP scores across dental status indicators (caries prevalence, gingival inflammation, dental infectious processes, number of posterior functional units) [29]. It was hypothesized that patients with a poorer dental status would have lower COHIP scores. Discriminant validity analysis was used, among other things, to assess the impact of oral diseases on children, thus addressing the secondary objective of the study. The COHIP score was also supposed to vary depending on different socio-demographic characteristics (region, gender, ethnicity, school, health insurance, place of living) and oral health behaviours (tooth-brushing frequency). Multilevel mixed models were used taking into account the cluster (school and dentist/examiner) effects.

A sensitivity analysis was performed to measure the impact of missing data on the results. First, we compared the socio-demographic characteristics of the children who responded completely to the COHIP-SF-19 to those who responded partially or were excluded due to non-responses. Then, to verify the representativeness of the sample, COHIP scores were weighted (per region, sex, and school type) to check the impact of participation rate on the scores. Moreover, descriptive statistics and internal consistency were both calculated in the main study group (at least 75% of the questions completed per domain) and in the group of children who answered completely to the COHIP questionnaire.

Data analyses were conducted using SPSS & AMOS software (IBM, Version 26) and Stata software (version 1.6, Stat/IC, StataCorp, College Station, US). The significance level was set at *p* < 0.05.

## Results

### Description of the population

**Fig. 1:**
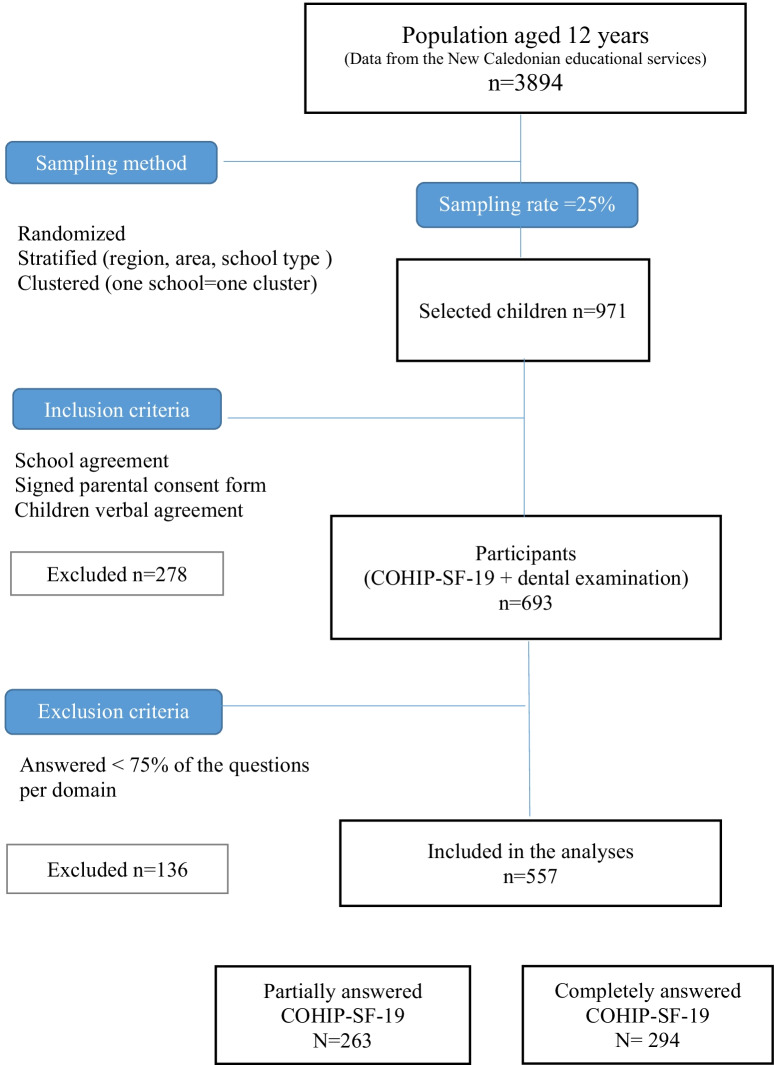
Description of the sample

A total of 693 children answered completely or partially the COHIP-SF-19 questionnaire, which correspond to a 75% participation rate. However, 136 children completed less than 75% of the questions per domain and were therefore excluded from the analyses. Thus, 557 children were included in the main analyses. The flow chart (Fig. 1) gives the number of children at each stage of the study process.

The socio-demographic profiles of the children is presented in Table 1. Mean age was 12.1 years ± 0.3, 75% of the children attended public schools and the proportion of girls was 53%. Self-reported ethnicity revealed that 29.4% of the children identified themselves as Oceanian, 7.7% as European, 46.5% with mixed belonging and 16.4% declared other ethnic origins (Caledonian, Asian…). Thirteen percent of the children lived in the Islands area, 20% in the Northern region and 67% in the Southern region. In NC, the whole population has access to partial dental coverage through the national public health fund system. Approximately 24% of children were benefiting from a supplementary public dental coverage for low income families, 56.4% had a private health insurance, while 19.3% had no supplementary health coverage. The majority of children (63%) lived in a town, a village or an isolated property, 35% in a tribe and 2% reported living in squats. The sensitivity analysis confirmed that the sociodemographic profile did not vary depending on the level of completion of the COHIP questionnaire (Table 1).

**Table 1:** Socio-demographic characteristics of the sample

	Whole sample	Answered < 75% per domain	Answered > 75% per domain (n = 557)			*p*^b^
			Partially answered	completely answered	*p*^a^	
	n = 693	n = 136	n = 263	n = 294		
Gender					0.9	0.44
Male	321 (46.3%)	59 (43.4%)	123 (46.8%)	139 (47.3%)		
Female	372 (53.7%)	77 (56.6%)	140 (53.2%)	155 (52.7%)		
Region					0.06	0.09
South	459 (66.2%)	84 (61.8%)	167 (63.5%)	208 (70.8%)		
North	137 (19.8%)	25 (18.4%)	54 (20.5%)	58 (19.7%)		
Islands	97 (14%)	27 (19.8%)	42 (16%)	28 (9.5%)		
Ethnicity					0.91	0.27
Oceanian	210 (30.8%)	49 (36.3%)	78 (30.2%)	83 (28.6%)		
European	49 (7.2%)	7 (5.2%)	18 (7%)	24 (8.3%)		
Multiracial	309 (45.2%)	54 (40%)	121 (46.9%)	134 (46.2%)		
Others	115 (16.8%)	25 (18.5%)	41 (15.9%)	49 (16.9%)		
Place of living					0.29	0.15
Tribe/Squat	261 (37.9%)	58 (43.3%)	102 (38.9%)	101 (34.59%)		
Town/village/property	427 (62.1%)	76 (56.7%)	160 (61.1%)	191 (65.4%)		
Type of school					0.8	0.42
Private	166 (23.95%)	29 (21.3%)	66 (25.1%)	71 (24.15%)		
Public	527 (76.05%	107 (78.7%)	197 (74.9%)	223 (75.85%		
Health insurance					0.5	0.71
Basic insurance only	133 (19.9%)	29 (22.3%)	46 (17.8%)	58 (20.7%)		
State aid supplemental	160 (23.9%)	29 (22.3%)	68 (26.2%)	63 (22.5%)		
Private supplemental	376 (56.2%)	72 (55.4%)	145 (56%)	159 (56.8%)		

^a^test Khi2, partially answered versus completely answered the COHIP questionnaire, for children who answered at least 75% of the questions per domain

^b^test Khi2, answered less than 75% of the questions per domain versus answered more than 75%

Oral diseases were frequent in the study population: 40% of the children had at least one decayed, missing or filled permanent tooth (DMFT > 0). Gingival inflammation was found in 45% of children, and 8% had at least one oral infectious process. About half of the children reported brushing their teeth twice a day, 38% once a day and 8% did not brush or did so occasionally. The dental status and oral health behaviours of the children are summarised in Table 2.

**Table 2:** Prevalence of oral diseases and frequency of tooth-brushing (n = 557)

Oral status	n (%)	[95% CI]
Dental caries		
% with DMFT = 0	334 (60%)	[56.4; 64.2]
% with DMFT > 0	223 (40%)	[35.8; 43.6]
Gingival status		
No gingivitis	252 (45.4%)	[41.2; 49.7]
Gingivitis (≥ 1 sextant)	303(54.6%)	[50.3; 58.8]
Oral infectious process		
No infectious process	511 (92.1%)	[90; 94.2]
Infectious process (≥ 1)	44 (7.9%)	[5.8; 10]
Number of PFU		
< 6	76 (13.6%)	[10.9; 16.5]
≥ 6	481 (86.4%)	[83.5; 89.1]
Tooth-brushing		
No/occasional brushing	46 (8.3%)	[6.1; 10.6]
Once a day	208 (37.5%)	[33.2; 41.6]
Twice a day	301 (54.2%)	[50.1; 58.7]

n, number; %, percentage; 95% CI, 95% confidence interval

### OHRQOL of the New Caledonian children

According to the COHIP questionnaire, 96.4% of the children experienced oral health problems since the beginning of the school year, 81.7% declared functional problems, and 90.5% had socio-emotional impacts (Table 3). Depending on the item, the percentage of non-respondents varied from 0.5% to 15%. The most common unanswered question was question 8 (feeling reassured). Additional file 4: Figure S1 illustrates the distribution of the responses and non-responses per item and per domain.

**Table 3:** Frequency distribution (n (%)) of the responses to COHIP-SF-19 questionnaire (n = 557)

Since school-year started	Almost always	Quite often	Sometimes	Almost never	Never
	n (%)	n (%)	n (%)	n (%)	n (%)
Domain 1: Oral health					
Q1: Had pain in your teeth/toothache (n = 550)	5 (0.9%)	28 (5.1%)	178 (32.4%)	129 (23.4%)	210 (38.2%)
Q2: Had discoloured teeth or spots on your teeth (n = 509)	24 (4.7%)	46 (9%)	98 (19.3%)	53 (10.4%)	288 (56.6%)
Q3: Had crooked teeth, paces between your teeth (n = 516)	40 (7.7%)	50 (9.7%)	93 (18%)	68 (13.2%)	265 (51.4%)
Q4: Had bad breath (n = 506)	23 (4.6%)	35 (6.9%)	193 (38.1%)	135 (26.7%)	120 (23.7%)
Q5: Had bleeding gums (n = 549)	39 (7.1%)	78 (14.2%)	152 (27.7%)	78 (14.2%)	202 (36.8%)
Domain 2: Functional well-being					
Q9: Had difficulty eating foods I would like to eat (n = 548)	28 (5.1%)	40 (7.3%)	96 (17.5%)	69 (12.6%)	315 (57.5%)
Q12: Had trouble sleeping (n = 552)	15 (2.7%)	20 (3.6%)	74 (13.4%)	55 (10%)	388 (70.3%)
Q15: Had difficulty saying certain words (n = 553)	10 (1.8%)	16 (2.9%)	50 (9.0%)	54 (9.8%)	423 (76.5%)
Q19: Had difficulty keeping your teeth clean(n = 537)	29 (5.4%)	56 (10.4%)	133 (24.8%)	105 (19.6%)	214 (39.9%)
Domain 3: Socio-emotional well-being					
Q6: Been unhappy or sad (n = 541)	15 (2.8%)	31 (5.7%)	64 (11.8%)	46 (8.5%)	385 (71.2%)
Q10: Felt worried or anxious (n = 546)	16 (2.9%)	26 (4.8%)	59 (10.8%)	84 (15.4%)	361 (66.1%)
Q11: Avoided smiling, laughing with other children (n = 553)	21 (3.8%)	33 (6.0%)	46 (8.3%)	53 (9.6%)	400 (72.3%)
Q16: Felt that you look different (n = 556)	13 (2.3%)	12 (2.2%)	52 (9.4%)	50 (9.0%)	429 (77.2%)
Q18: Been worried about what other people think (n = 545)	21 (3.9%)	19 (3.5%)	63 (11.6%)	71 (13.0%)	371 (68.1%)
Q14: Teased, bullied, called names by other children (n = 554)	9 (1.6%)	15 (2.7%)	35 (6.3%)	46 (8.3%)	449 (81.1%)
Q7: Missed school (n = 548)	7 (1.3%)	20 (3.6%)	54 (9.9%)	56 (10.2%)	411 (75.0%)
Q13: Did not want to speak/read out loud in class (n = 552)	11 (2.0%)	8 (1.5%)	36 (6.5%)	31 (5.6%)	466 (84.4%)
Q8: Been reassured or put in trust through (n = 472)	92 (19.5%)	56 (11.9%)	97 (20.5%)	57 (12.1%)	170 (36.0%)
Q17: Felt that you were good looking (n = 511)	34 (6.7%)	27 (5.3%)	89 (17.4%)	60 (11.7%)	301 (58.9%)

n, number; %, percentage

The COHIP scores ranged from 7 to 76, with a mean score of 57.87 ± 9.98. The means, ranges, quartiles of the sub-scores and the total score of the COHIP-SF-19 are presented in Table 4. No floor or ceiling effects were found for the total score (Table 4). In the functional well-being domain, a ceiling effect was observed indicating that extreme items were missing in the upper end of this sub-scale.

**Table 4:** Descriptive statistics for the COHIP-SF-19 scores (n = 557)

	Domain 1: Oral health	Domain 2: Functional well-being	Domain 3: Socio-emotional well-being	COHIP-SF 19 total score
Mean score (SD)	14.07 ± 3.73	12.86 ± 2.77	30.94 ± 5.75	57.9 ± 9.96
Weighted mean score^a^ (SD)	13.91 ± 3.87	12.76 ± 2.9	30.91 ± 5.88	57.6 ± 10.41
Range	3–20	3–16	0–40	7–76
Proportion of lowest possible score	0.7%	0.4%	0.2%	0.2%
Proportion of highest possible score	4.8%	19.7%	3.1%	0.4%
1st quartile	12	11	28	52
3rd quartile	17	15	35	65

^a^Weighted per region, sex, and school type to take into account participation rate

### Internal consistency

Cronbach’s alpha for the global COHIP-SF-19 score was 0.802 and increased slightly if the two items with the lowest item-rest correlations (Q8, Q17) were deleted. Cronbach’s alphas for subscales values were as follows: Oral Health = 0.624; Functional Well-Being = 0.495; Socio- emotional Well-Being = 0.703. The Cronbach’s alpha, “alpha if item deleted” and item-rest correlation, for each domain and for the overall COHIP-SF-19 are shown in Table 5. The item-rest correlations for the items Q8 and Q17 were below the recommended threshold of 0.2 [30].

**Table 5:** Internal reliability for the French COHIP-SF-19 questionnaire (n = 557)

Since school-year started	Cronbach alpha	Item-rest correlation	Total Cronbach’s alpha if item delated
Domain 1: Oral health	0.624		
Q1: Had pain in your teeth/toothache		0.47	0.789
Q2: Had discoloured teeth or spots on your teeth		0.403	0.792
Q3: Had crooked teeth, paces between your teeth		0.369	0.794
Q4: Had bad breath		0.371	0.794
Q5: Had bleeding gums		0.291	0.8
Domain 2: Functional well-being	0.495		
Q9: Had difficulty eating foods I would like to eat		0.448	0.789
Q12: Had trouble sleeping		0.52	0.786
Q15: Had difficulty saying certain words		0.374	0.794
Q19: Had difficulty keeping your teeth clean		0.299	0.799
Domain 3: Socio-emotional well-being	0.703		
Q6: Been unhappy or sad		0.532	0.784
Q10: Felt worried or anxious		0.504	0.786
Q11: Avoided smiling, laughing with other children		0.556	0.783
Q16: Felt that you look different		0.502	0.788
Q18: Been worried about what other people think		0.412	0.791
Q14: Teased, bullied, called names by other children		0.495	0.790
Q7: Missed school		0.423	0.792
Q13: Did not want to speak/read out loud in class		0.502	0.789
Q8: Been reassured or put in trust through		0.099	0.817
Q17: Felt that you were good looking		0.032	0.819
COHIP-SF-19 total score	0.802		

### Confirmatory factor analysis

**Fig. 2:**
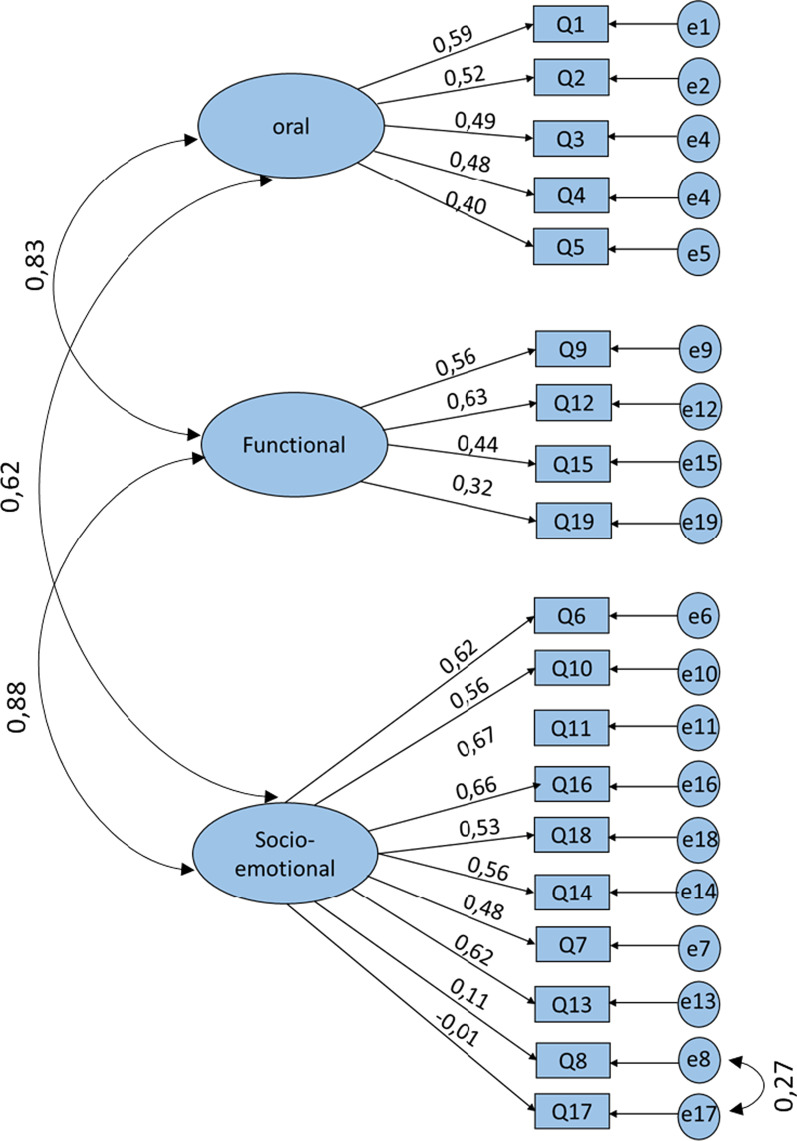
Confirmatory factorial analysis with the original three dimensions COHIP-SF19 model (Model 1)

Model 1: The three-factor model maintains the same structure as the original COHIP-SF-19. This model showed inter-factor correlation coefficients ranging from 0.62 to 0.88. Two items, Q8 and Q17, had small factor loadings < 0.1 (Fig. 2).

**Fig. 3:**
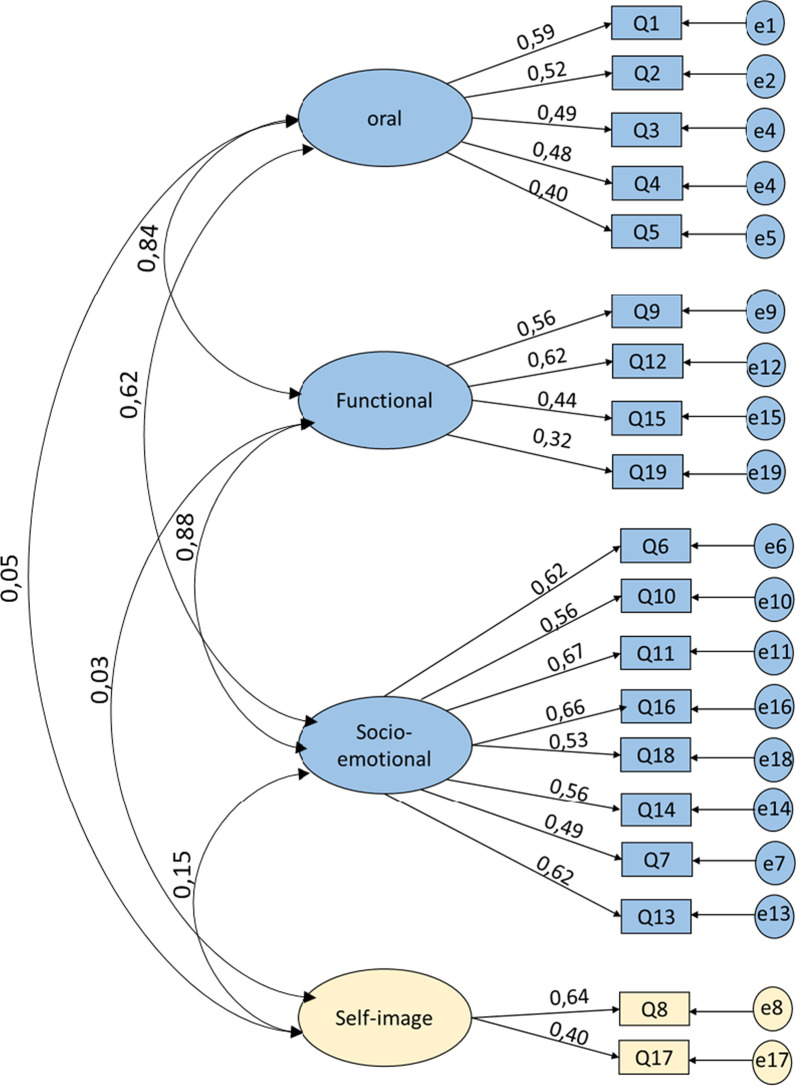
Confirmatory factorial analysis with a proposed four dimensions COHIP-SF19 model (Model 2)

Model 2: The four-factor model emerges from the CFA and EFA results of previous studies evaluating the structure of the original model [24, 25, 31]. In this model, questions Q8 and Q17 were extracted as a new factor. This model provided better factor loadings in particular for Q8 and Q17. It should be noted that these two questions belonged to the « self-image» subscale in the original version (COHIP-34). The inter-factor correlation coefficients between the three original factors remained unchanged. However, a low correlation coefficient was observed with the new factor (Fig. 3).

The CFA indicated that Model 1 (original three-factor model) and 2 (four-factor model) did not provide an acceptable fit according to the recommended standards; χ2/df > 3, AGFI < 0.85,CFI < 0.9. The RMSA values were also slightly below 0.08 (Table 6) [26].

**Table 6:** Factorial analyses: Comparison of the adjustment measures for the three models evaluated

Models^a^	*p*	X^2^/DF	RMSEA	GFI	AGFI	CFI	AIC
Model 1^b^	0.001	3.751	0.07	0.892	0.861	0.724	639.134
Model 2^c^	0.001	3.779	0.071	0.892	0.860	0.823	641.462
Model 3^d^	0.001	2.731	0.056	0.926	0.905	0.889	488.123
Good fit	[0.05; 1]	[0; 2]	[0; 0.05]	[0.95; 1]	[0.9; 1]	[0.97; 1]	> AIC
Acceptable	[0.01; 0.05]	[2; 3]	[0.05; 0.08]	[0.9; 0.95]	[0.85; 0.9]	[0.95; 0.97]	> AIC

DF, degrees of freedom; X2, Khi2 value; *p*, *p* value; RMSEA, root mean square error of approximation; GFI, Goodness of Fit Index; CFI, Comparative Fit Index; AGFI, Adjusted Goodness of Fit Index; AIC, Akaike Information Criterion

^a^CFA: confirmatory factorial analysis

^b^Model 1: CFA with the original three dimensions COHIP-SF19 model

^c^Model 2: CFA with the proposed four dimensions COHIP-SF19 model

^d^Model 3: CFA with a modified three dimensions model using Modification Indices

> AIC: > to Akaike Information criterion (AIC) for comparison model

**Fig. 4:**
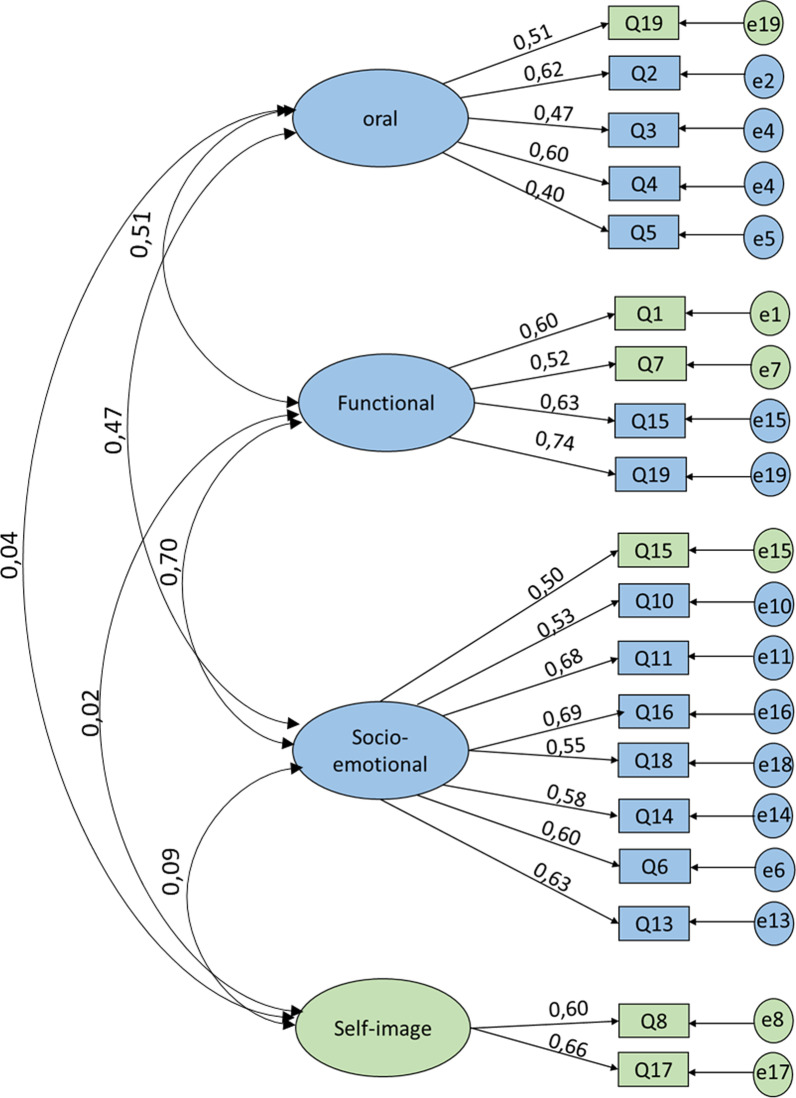
Confirmatory factorial analysis with a modified three dimensions model using Modification Indices (Model 3)

Model 3: The analysis of the variation in factor loadings between models 1 and 2, and the modification indices (MI) were used to find potential sources of significant model improvement. Only the more important MIs were considered as indicators of model improvement [32]. This analysis showed a strong association between questions Q1, Q7 and domain 2 (functional well-being), between question Q19 and domain 1 (oral health) and between question Q15 and domain 3 (socio-emotional well-being). The fit indices of model 3 indicated an overall adequate-good model fit (χ2/df > 3, RMSA = 0.056, GFI & AGFI > 0.9). In addition, this model has a lower AIC value than models 1 and 2 (Table 6) [26]. Overall, Model 3 has better factor loadings, especially for questions Q4, Q9, Q15, Q17 and Q19. However, factor loadings for questions Q3, Q6 and Q10 have decreased slightly, but remained acceptable (> 0.4) (Fig. 4).

### Reproducibility

The test–retest reliability of the overall COHIP was excellent (ICC = 0.90; *p* < 0.0001). At the level of subscales, test–retest reliability was good for the oral health subscale and excellent for the functional and socio-emotional well-being subscales, with respectively ICCs values of 0.791; 0.873 and 0.892 (*p* < 0.0001).

### Concurrent validity

Results for the concurrent validity are presented in Tables 7 and 8. As expected, lower scores were found for children with severe oral health problems and who reported difficulties in accessing oral health care. The calculated effect sizes were high for self-perceived oral health problems and moderate to small (domain 1) for the perception of oral health care access difficulties. Correlation coefficients between COHIP scores and these variables were low, but negative and significant (*p* < 0.001). When considering the subscales, it appeared that the highest correlation was found between the perception of dental problems and the oral health dimension (Table 8).

**Table 7:** Concurrent validity: COHIP-SF-19 scores and self-perceived problems (n = 557)

	Domain 1: Oral health	Domain 2: Functional well-being	Domain 3: Socio-emotional well-being	COHIP-SF 19 total score
Oral health problems				
No (n = 267)	15.39 ± 3.17	13.61 ± 2.52	32.85 ± 4.55	61.85 ± 7.69
A few (n = 261)	12.92 ± 3.7	12.31 ± 2.73	29.57 ± 5.89	54.80 ± 9.91
Many/A lot (n = 28)	12.32 ± 4.65	10.71 ± 3.59	25.39 ± 7.41	48.43 ± 13.54
*p*^a^	< 0.001	< 0.001	< 0.001	< 0.001
Effect size^b^ [95% IC]	0.92 [0.53–1.32]	1.10 [0.70–1.49]	1.53 [1.12–1.94]	1.60 [1.19–2.00]
Access to oral health care				
No difficulty (n = 380)	14.65 ± 3.45	13.28 ± 2.5	31.89 ± 5.1	59.82 ± 8.83
Difficulties (n = 175)	12.84 ± 4.01	11.91 ± 3.1	28.86 ± 6.56	53.61 ± 11.04
*p*^a^	< 0.001	< 0.001	0.01	< 0.001
Effect size [95% IC]	0.48 [0.30–0.67]	0.51 [0.32–0.69]	0.54 [0.36–0.72]	0.64 [0.46–0.83]

^a^*p* values, Kruskal Wallis test

^b^No vs many/A lot

**Table 8:** Concurrent validity: correlation between COHIP-SF-19 scores and self-perceived problems (n = 557)

Spearman correlation coefficient	Oral health problems	Difficulty in accessing dental care
Domaine 1: Oral health	− 0.414*	− 0.216*
Domain 2: Functional well-being	− 0.331*	− 0.218*
Domain 3: Socio-emotional well-being	− 0.295*	− 0.240*
COHIP-SF-19 total score	− 0.357*	− 0.286*

*p < 0.001

### Known groups and discriminant validity

The relationships between COHIP-SF-19 scores and various socio-demographic, behavioural and clinical variables are presented in Table 9. The COHIP-SF-19 scores varied significantly related to gender, place of living, ethnic group, type of school, health coverage and the region. Children with at least one decayed, missing or filled permanent tooth (D_3_MFT > 0) and children with infectious processes experienced higher OHRQoL impacts. The results showed that COHIP-SF-19 scores were significantly lower among children with less optimal clinical status. No significant variation was observed depending on the number of functional units nor the presence of gingival inflammation. Children who reported brushing twice a day had significantly higher COHIP-SF-19 scores than those who brushed more occasionally.

**Table 9:** Discriminant validity: COHIP-SF-19 scores and socio-demographic, behavioural, clinical variables (whole sample, n = 557)

Variables	Domain 1: Oral health			Domain 2: Functional well-being			Domain 3: Socio-emotional well-being			Total score		
	Mean (SD)	RC [IC]	*p*	Mean (SD)	RC [IC]	*p*	Mean (SD)	RC [IC]	*p*	Mean (SD)	RC [IC]	*p*
Gender												
Male (262)	14.45 ± 3.84			13.12 ± 2.65			31.46 ± 5.31			59.00 ± 9.49		
Female (295)	13.73 ± 3.60	− 0.05 [− 0.1; 0.03]	**0.04**	12.62 ± 2.88	− 0.05 [− 0.09; − 0.003]	**0.03**	30.49 ± 6.07	− 0.03 [− 0.07; 0.007]	**0.05**	56.84 ± 10.31	− 0.04 [− 0.08; − 0.01]	**< 0.01**
Region												
South (375)	14.55 ± 3.5			13.11 ± 2.62			31.45 ± 5.45			59.12 ± 9.35		
North (112)	13.91 ± 3.4	− 0.02 [− 0.1; 0.07]	0.61	12.81 ± 2.69	− 0.02 [− 0.08; 0.04]	0.55	30.83 ± 4.81	− 0.01 [− 0.06; 0.03]	0.50	57.56 ± 8.35	− 0.02 [− 0.07; 0.03]	0.38
Islands (70)	11.73 ± 4.5	− 0.2 [− 0.36; − 0.14]	**< 0.01**	11.56 ± 3.39	− 0.15 [− 0.22; − 0.07]	**< 0.01**	28.37 ± 7.73	− 0.1 [− 0.16; − 0.05]	**< 0.01**	51.66 ± 13.01	− 0.17 [− 0.22; − 0.11]	**< 0.01**
Ethnicity												
Oceanian (161)	13.17 ± 3.95			12.13 ± 2.99			30.08 ± 6.15			55.38 ± 10.8		
European (42)	16.36 ± 2.82	0.18 [0.07; 0.29]	**< 0.01**	14.26 ± 1.82	0.17 [0.07; 0.26]	**< 0.01**	32.71 ± 5.42	0.09 [0.02; 0.16]	**0.01**	63.33 ± 7.83	0.12 [0.04; 0.19]	**< 0.01**
Multiracial (255)	14.07 ± 3.53	0.07 [0.004; 0.13]	**0.04**	12.86 ± 2.77	0.06 [0.01; 0.12]	**0.02**	30.88 ± 5.47	0.03 [− 0.01; 0.7]	0.13	57.81 ± 9.49	0.04 [0.009; 0.08]	**0.04**
Others (90)	14.60 ± 3.63	0.07 [− 0.01; 0.15]	**0.09**	13.38 ± 2.55	0.1 [0.03; 0.17]	**0.004**	31.72 ± 5.92	0.08 [0.02; 0.13]	**< 0.01**	59.7 ± 9.56	0.06 [0.001; 0.11]	**0.04**
Place of living												
Tribe/Squat (203)	13.16 ± 3.81			12.01 ± 3.13			29.9 ± 6.16			55.08 ± 10.65		
Town/village (351)	14.58 ± 3.58	0.54 [− 0.01; 0.12]	0.1	13.32 ± 2.45	0.12 [0.07; 0.17]	**< 0.01**	31.48 ± 5.4	0.06 [0.02; 0.1]	**< 0.01**	59.38 ± 9.17	0.06 [0.02; 0.10]	**< 0.01**
Type of school												
Private (137)	13.53 ± 4.05			12.61 ± 2.93			30.63 ± 6.42			56.76 ± 11.36		
Public (420)	14.25 ± 3.61	0.01 [− 0.1; 0.12]	0.85	12.93 ± 2.74	0.01 [− 0.06; 0.08]	0.79	31.04 ± 5.51	0.004 [− 0.04; 0.05]	0.86	58.23 ± 9.48	0.01 [− 0.06; 0.08]	0.76
Health insurance												
State aid (131)	13.15 ± 4.04			11.96 ± 3.06			29.48 ± 6.6			54.59 ± 11.16		
Basic insurance only (104)	13.85 ± 3.62	0.50 [− 0.03; 0.13]	0.24	12.67 ± 2.71	0.06 [− 0.002; 0.14]	0.058	31.30 ± 5.18	0.06 [0.006; 0.12]	**0.03**	57.82 ± 9.66	0.06 [0.004; 0.12]	**0.04**
Private supplemental (304)	14.45 ± 3.56	0.72 [0.004; 0.14]	**0.04**	13.31 ± 2.58	0.1 [0.05; 0.16]	**< 0.01**	31.39 ± 5.53	0.06 [0.02; 0.10]	**< 0.01**	59.14 ± 9.34	0.07 [0.03; 0.12]	**< 0.01**
Toothbrushing												
No/occasional (46)	12.15 ± 3.63			11.87 ± 3.26			28.98 ± 6.93			53. ± 11.33		
Once a day (208)	13.64 ± 3.72	0.05 [− 0.04; 0.16]	0.29	12.43 ± 2.71	0.04 [− 0.38; 0.13]	0.27	30.54 ± 5.4	0.07 [0.03; 0.14]	0.04	56.61 ± 9.43	0.05 [− 0.02; 0.11]	0.18
Twice a day (301)	14.67 ± 3.62	0.11 [0.007; 0.21]	**0.03**	13.30 ± 2.69	0.11 [0.02; 0.19]	**0.01**	31.52 ± 5.72	0.1 [0.04; 0.17]	**< 0.01**	59.49 ± 9.83	0.08 [0.01; 0.15]	**0.02**
DMFT												
DMFT = 0 (334)	14.70 ± 3.42			13.37 ± 2.39			31.68 ± 5.24			59.75 ± 8.66		
DMFT > 0 (223)	12.99 ± 3.93	− 0.11 [− 0.17; − 0.06]	**< 0.01**	12.10 ± 3.13	− 0.11 [− 0.15; − 0.06]	**< 0.**01	29.76 ± 6.38	− 0.06 [− 0.10; − 0.02]	**< 0.01**	54.85 ± 11.17	− 0.09 [− 0.12; − 0.05]	**< 0.01**
Gingival status												
No gingivitis (252)	14.21 ± 3.69			13.04 ± 2.74			31.21 ± 5.51			58.46 ± 9.67		
Gingivitis (> 1sextant) (303)	13.95 ± 3.75	− 0.05 [− 0.1; 0.1]	0.11	12.74 ± 2.79	− 0.44 [− 0.09; 0.003]	0.07	30.77 ± 5.92	− 0.01 [− 0.52; 0.02]	0.43	57.47 ± 10.16	− 0.03 [− 0.07; 0.003]	0.07
Infectious process												
No (511)	14.26 ± 3.57			13.06 ± 2.64			31.30 ± 5.48			58.62 ± 9.27		
At least one (44)	11.42 ± 4.45	− 0.26 [− 0.35; − 0.16]	**< 0.01**	10.77 ± 3.21	**-**0.2 [− 0.28; − 0.12]	**< 0.01**	26.93 ± 7.39	− 0.13 [− 0.19; − 0.06]	**< 0.01**	49.12 ± 13.24	− 0.2 [− 0.27; − 0.14]	**< 0.01**
Number of PFU												
< 6 (76)	14.29 ± 3.88			12.74 ± 2.81			30.3 ± 6.28			57.31 ± 10.76		
≥ 6 (481)	14.04 ± 3.71	− 0.02 [− 0.08; 0.74]	0.96	12.87 ± 2.79	0.02 [− 0.04; 0.09]	0.52	31.05 ± 5.66	0.03 [− 0.17; 0.08]	0.18	57.96 ± 9.86	0.02 [− 0.03; 0.07]	0.40

Significant values are in bold

*p Multilevel mixed-effects linear regression, RC regression coefficient

### Sensitivity analyses

The psychometric properties of COHIP-SF-19 scale were also assessed for the group of children who answered completely to the COHIP questionnaire (Additional file 2: Table S2 and Additional file 3: Table S3). Interestingly, sensitivity analyses showed similar results as compared to the main analyses in which missing data were imputed. Some few variations can be noticed in the group of complete responders (n = 294) such as for a higher low-range for the total score (23 instead of 7). Cronbach alpha were similar in both groups.

## Discussion

The aim of this study was to validate the French version of the COHIP-SF-19, from a sample of 12-year-old children in New Caledonia in 2019. The validation of such a scale is essential for the monitoring of OHRQoL in school-aged children and for evaluating the impact of oral health promotion programs. Since the full version of the French COHIP-SF-34 has already been evaluated in a previous study, the validation procedure did not include a translation and trans-cultural adaptation step [10]. The results of the present study showed that the psychometric characteristics of the French COHIP-SF-19 were satisfactory, which could allow its future use in France.

The mean scores of the COHIP-SF-19 observed among the 12-year-old school children in NC in 2019/2020 were relatively low as compared to those found in some other COHIP-SF-19 validation studies [24, 25, 33–35]. Particularly, a survey conducted in Libya in 2016 among 12-years old children demonstrated very high levels of OHrQOL while caries prevalence was similar [24]. Thus, children in NC perceived high impacts of oral disease on their everyday life. These differences between countries might indicate that the subjective perception of oral health vary depending on geographical, social or cultural aspects, and not only on dental status.

In our study, the overall score and sub-scores of the COHIP-SF-19 were lower for girls than for boys. In contrast, higher scores for girls were found in the Japanese COHIP-SF-19 validation study [25]. In terms of general health-related quality of life (HRQoL), adolescent girls tend to have a lower score than adolescent boys. This may be related to the more significant physical changes during puberty for girls than for boys [36].

Reliability was adequate with Cronbach alpha values close to those reported in the literature (= 0,80). At the sub-scale level, only the socio-emotional well-being sub-scale showed an acceptable Cronbach's alpha value. This finding also is reported for the Arabic, Japanese and Chinese versions of the COHIP-SF-19 [24, 25, 33]. The Cronbach's alpha values for the oral health and functional well-being domains were relatively low. As suggested in previous studies, this is probably related to the small number of items that compose them [25, 37].

The evaluation of discriminant validity showed that the COHIP-SF-19 was able to differentiate children with different behavioural, social and clinical status. However, gingivitis scores and the number of posterior functional units were not associated to the COHIP-SF-19 scores. The impacts of gingivitis on OHrQoL might be limited as already suggested in the Japanese validation study [25].

COHIP scores differed according to the social status such as ethnicity, region of residence, place of living and health insurance coverage. These variations are in line with social oral health disparities that have previously been pointed out in the 2012 study. Region of residence and ethnicity had been identified as major social determinants allowing identification of high risk groups such as native Oceanian children living in the Islands province [14, 38]. It is thus not surprising to observe lower COHIP-SF-19 scores in Oceanian children and/or children living in the Islands and even the North Provinces. These results underline the concept of social determinants of health within which the region of residence, place of living and ethnicity are key inter-related social factors.

Concurrent validity was demonstrated with significant relationships between the COHIP-SF-19 scores and self-perceived oral health problems or difficulties in accessing oral health care. The oral health sub-scale was strongly correlated to the perception of dental problems. These findings are in agreement with previous studies [25, 33].

The factor structure of the French COHIP-SF-19 was examined using confirmatory factor analyses (CFA) [23]. The transfer of some items into other domains, as compared to the original structure, can be interpreted specifically within the Caledonian context. Indeed, children in NC associated Q19 (difficulties in keeping teeth clean) with the concept of oral health, which could be related to the implementation of the oral health promotion program in NC. The association between Q7 (Missed school) and domain 2 (Functional well-being) could represent the perception that school attendance is a function for children, along with eating and sleeping. Dental pain affects oral function, leading to difficulties in eating or sleeping. Therefore, children could be more likely to associate Q1 (Having a toothache) with the functional well-being domain. Finally, speech is a function that allows interaction with others, which may explain why Q15 (difficulty saying certain words) was associated to the socio-emotional well-being domain. To confirm these hypotheses, further studies are needed in different contexts to better understand children's representations of these items and the influence of local culture on their representations.

Questions 8 and 17 were the most common unanswered questions and showed the smallest factor loadings in the CFA of model 1. These results have also been reported in other validation studies of the COHIP-SF-19 [24, 25, 35]. It should be noted that these two items are the only positively worded questions, which could potentially lead to confusion [25]. Furthermore, questions 8 and 17 were initially grouped along with four other questions in a separate subscale (self-image) in the COHIP-SF-34. Our CFA results showed that the inclusion of these two items in the socio-emotional well-being subscale was not entirely appropriate and it was proposed to put them aside, which improved the structure of the scale. These hypotheses would also need to be further explored by considering the properties of the new structure (model 3) within different populations.

One of the strengths of this study is the use of a large sample regrouping children from various cultural, geographical and social profiles within New Caledonia. The number of children included in the analyses was large (> 500) in accordance with COSMIN guidelines, thus guarantying a satisfactory statistical power, namely for evaluating the internal validity. Moreover, the impact of non-participation and partial responding to the COHIP questionnaire was also checked through sensitivity analyses. These indicated very few variations as compared to the main results.

This study had some limitations. First, the concurrent validity did not integrate a variable that directly assessed “self-perceived oral health”. In addition, for the assessment of reproducibility, intra-class correlation coefficients (ICCs) were calculated using data from the 2012 study that allowed the validation of the French COHIP-34. Due to the COVID-19 pandemic, it has been impossible to get a second round of data at the beginning of year 2020 for a specific COHIP-19 test–retest evaluation. Moreover, sensitivity to change was not evaluated in our study. Earlier studies have already assessed the ability of the English COHIP-SF-19 to detect changes over time, showing a "none to moderate" sensitivity that will need to be confirmed [39, 40]. External validity can also be questioned as New Caledonia represents a very specific cultural, ethnic and social context. The generalization of our results to all French speaking countries or regions would need to be confirmed. However, coherence with the results of similar studies as well as the social characterization of the study sample may support the use of the COHIP-short form within other populations or contexts.

The advantage of COHIP-SF-19 is that the questionnaire requires less time as compared to the original 34-item version or other longer instruments such as the 37-items of the CPQ11-14 [6, 9]. In addition, the COHIP-SF-19 can be applied to a wider age range; the age group has been extended to 7–18 years in recent publications, while the short form of CPQ11-14 (8–16 items) is assessing OHRQoL of children aged 11–14 years [41]. Therefore, the validation of the French version of the COHIP-SF19 offers many opportunities for researchers to compare epidemiological and clinical situations of different populations worldwide.

## Conclusion

The aim of this study was to validate the French version of the COHIP-SF-19, from a sample of 12 years old children in NC in 2019/2020. The results showed that the psychometric characteristics were satisfactory which could allow its use in the future in France. This study also proposed some improvement of the factor structure. It must be noticed that the COHIP-SF-19 scores reflected the social gradient of health among New Caledonian children. OHRQoL research is essential for oral health care planning and for the implementation of public policies, as it can help direct interventions to the most impacted populations.

## Supplementary Information

**Additional file 1: Table S1.** Description of the study variables

**Additional file 2: Table S2.** Descriptive statistics for the COHIP-SF-19 scores for children who completely answered the questionnaire (n = 294)

**Additional file 3: Table S3.** Internal reliability for the French COHIP-SF-19 questionnaire for children who completely answered the questionnaire (n = 294)

**Additional file 4: Figure S1.** Frequency distribution (%)) of the responses to COHIP-SF-19 questionnaire per domain (n = 557)

**Additional file 5.** COSMIN Risk of Bias checklist

## Data Availability

The data that support the findings of this study are openly available in “zenodo” at https://doi.org/10.5281/zenodo.5876264. To protect the anonymity of the participating children, certain variables, which if crossed, could reveal the identity of the children, such as place of residence and type of school, were removed from the open database.
