# Supplementary material for: Validation of the French version of COHIP-SF-19 among 12-years children in New Caledonia
Source: BMC Oral Health. 2022 Aug 18;22:358. doi: 10.1186/s12903-022-02370-4 (PMC9387427; doi:10.1186/s12903-022-02370-4)
Supplement: Supplementary file 1 — Additional file 1: Table S1. Description of the study variables [file 12903_2022_2370_MOESM1_ESM.docx]

Supplementary Table 1: Description of the study variables

| Variables | Answer options | Source data | | |
| --- | --- | --- | --- | --- |
|  |  | School register | Self-administered questionnaire | Clinical examination |
| **Sociodemographic status, Ethnicity & conditions of living** | | | | |
| Gender | Male **;** Female |  |  |  |
| Region | South**;** North**;** Islands |  |  |  |
| Ethnicity | Oceanian**;** European**;** Multiracial**;** Other |  |  |  |
| Place of living | Tribe/squat **;** Town/village/Countryside |  |  |  |
| Health insurance | Basic public insurance only**;** State aid supplemental**;** Private supplemental |  |  |  |
| Type of school | Public; Private |  |  |  |
| **Dental status** | | | | |
| DMFT | DMFT =0 ; DMFT >0 |  |  |  |
| Gingival status | No gingivitis; Gingivitis > 1sextant |  |  |  |
| Infectious process | No ; At least one |  |  |  |
| Numer of Posterior Functional Units (PFU) | <6 ; =>6 |  |  |  |
| **Access to oral health care and prevention** | |  |  |  |
| Access to oral health care | No difficulties; Difficulties |  |  |  |
| **Oral health behaviours** | |  |  |  |
| Frequency of tooth brushing | Twice a day or more; Once a day or less |  |  |  |
| **Impacts of oral diseases on OHrQOL** | | | | |
| Perceived oral health problems | None; A few; Many |  |  |  |
| COHIP-SF 19 questionnaire | Q1 to Q19 |  |  |  |
