# Supplementary material for: Validation of the French version of COHIP-SF-19 among 12-years children in New Caledonia
Source: BMC Oral Health. 2022 Aug 18;22:358. doi: 10.1186/s12903-022-02370-4 (PMC9387427; doi:10.1186/s12903-022-02370-4)
Supplement: Supplementary file 2 — Additional file 2: Table S2. Descriptive statistics for the COHIP-SF-19 scores for children who completely answered the questionnaire (n = 294) [file 12903_2022_2370_MOESM2_ESM.docx]

| Supplementary Table S2 : Descriptive statistics for the COHIP-SF-19 scores for children who completely answered the questionnaire (n=294) | | | | |
| --- | --- | --- | --- | --- |
|  | **Domain 1**  **Oral health** | **Domain 2**  **Functional well-being** | **Domain 3**  **Socio-emotional well-being** | **COHIP-SF-19** **Total score** |
| Mean Score (Standard deviation) ^*^ | 14.31±3.64 | 13.07±2.73 | 30.93±5.14 | 58.32±9.34 |
| Range | 3-20 | 3-16 | 13-40 | 23-76 |
| Proportion of lowest possible score | 0.3% | 0.3% | 0.3% | 0.7% |
| Proportion of highest possible score | 5.8% | 22.1% | 1% | 0.7% |
| 1^st^ quartile | 12 | 11 | 29 | 52 |
| 3^rd^ quartile | 17 | 15 | 34 | 65 |
