# Supplementary material for: Validation of the French version of COHIP-SF-19 among 12-years children in New Caledonia
Source: BMC Oral Health. 2022 Aug 18;22:358. doi: 10.1186/s12903-022-02370-4 (PMC9387427; doi:10.1186/s12903-022-02370-4)
Supplement: Supplementary file 3 — Additional file 3: Table S3. Internal reliability for the French COHIP-SF-19 questionnaire for children who completely answered the questionnaire (n = 294) [file 12903_2022_2370_MOESM3_ESM.docx]

| Supplementary Table S3 : Internal reliability for the French COHIP-SF-19 questionnaire for children who completely answered the questionnaire (n=294) | | | |
| --- | --- | --- | --- |
| **Since school-year started** | **Cronbach alpha** | **Item-rest correlation per domain** | **Total Cronbach’s alpha if item delated** |
| **Domain 1 : Oral Health** | **0.57** |  |  |
| Q1 : Had pain in your teeth/toothache |  | 0.442 | 0.763 |
| Q2 : Had discoloured teeth or spots on your teeth |  | 0.37 | 0.767 |
| Q3 : Had crooked teeth, paces between your teeth |  | 0.354 | 0.769 |
| Q4 : Had bad breath |  | 0.335 | 0.769 |
| Q5 : Had bleeding gums |  | 0.305 | 0.773 |
| **Domain 2 : Functional Well-Being** | **0.49** |  |  |
| Q9 : Had difficulty eating foods I would like to eat |  | 0.445 | 0.761 |
| Q12 : Had trouble sleeping |  | 0.471 | 0.761 |
| Q15 : Had difficulty saying certain words |  | 0.385 | 0.767 |
| Q19 : Had difficulty keeping your teeth clean |  | 0.307 | 0.772 |
| **Domain 3 Socio-emotional Well-Being** | **0.67** |  |  |
| Q6 : Been unhappy or sad |  | 0.478 | 0.76 |
| Q10 : Felt worried or anxious |  | 0.415 | 0.764 |
| Q11 : Avoided smiling, laughing with other children |  | 0.521 | 0.756 |
| Q16 : Felt that you look different |  | 0.495 | 0762 |
| Q18 : Been worried about what other people think |  | 0.408 | 0.765 |
| Q14 : Teased, bullied, called names by other children |  | 0.42 | 0.766 |
| Q7 : Missed school |  | 0.363 | 0.769 |
| Q13 : Did not want to speak/read out loud in class |  | 0.516 | 0.763 |
| Q8 : Been reassured or put in trust through |  | 0.059 | 0.796 |
| Q17 : Felt that you were good looking |  | -0.019 | 0.797 |
|  |  |  |  |
| **COHIP-SF-19 Total score** | **0.78** |  |  |
