# Supplementary material for: Validation of the French version of COHIP-SF-19 among 12-years children in New Caledonia
Source: BMC Oral Health. 2022 Aug 18;22:358. doi: 10.1186/s12903-022-02370-4 (PMC9387427; doi:10.1186/s12903-022-02370-4)
Supplement: Supplementary file 4 — Additional file 4: Figure S1. Frequency distribution (%)) of the responses to COHIP-SF-19 questionnaire per domain (n = 557) [file 12903_2022_2370_MOESM4_ESM.docx]

Supplementary Figure S1: Frequency distribution (%)) of the responses to COHIP-SF-19 questionnaire per domain (n=557)
